# Bispecific antibodies targeting herpes simplex virus glycoproteins B and D

**DOI:** 10.3389/fimmu.2026.1803389

**Published:** 2026-05-11

**Authors:** Doina Atanasiu, Wan Ting Saw, Tina M. Cairns, Harvey M. Friedman, Gary H. Cohen

**Affiliations:** 1 Department of Basic and Translational Sciences, School of Dental Medicine, University of Pennsylvania, Philadelphia, PA, United States; 2 Infectious Disease Division, Department of Medicine, Perelman School of Medicine, University of Pennsylvania, Philadelphia, PA, United States; 3 Penn Institute for RNA Innovation, University of Pennsylvania, Philadelphia, PA, United States

**Keywords:** bispecific antibodies, glycoproteins, HSV, neutralizing antibodies, recombinant antibodies

## Abstract

**Background:**

Herpes simplex viruses (HSV-1 and HSV-2) can be transmitted both orally and sexually and cause lifelong morbidity and, in some cases, meningitis and encephalitis. Although several clinical trials using subunit antigens have been conducted, no vaccine exists. One problem in viral treatment is the emergence of drug resistance through mutations, thereby conferring resistance. Treatment with antibodies targeting multiple epitopes, such as antibody cocktails or bispecific antibodies (BsAbs), should reduce the likelihood of viral escape.

**Methods:**

We screened pairs of antibodies targeting key functional sites on glycoproteins B (gB) and D (gD), which are crucial for HSV entry. Murine hybridoma cells expressing anti-gD and anti-gB Mabs were sequenced. Recombinant antibodies, mono- and bispecific, were purified from supernatants of 293T transfected cells.

**Results:**

From a panel of gD-gB monoclonal antibodies (Mabs), we selected MC2 (anti-gD) and C226 (anti-gB) for the design of BsAbs: MC2/C226sc and a simpler version in which the C226scFv was cloned at the C-terminus of MC2 Fc, which we termed BD Tetra. Notably, both MC2/C226sc and BD Tetra BsAbs simultaneously recognized and bound gB and gD and had a marked synergistic effect on inhibiting cell-cell fusion, virus entry, and plaque formation. Although MC2 is HSV-2 specific, both MC2/C226sc and BD Tetra showed activity against HSV-1. The Fc domains of these two BsAbs were equally effective at binding to mouse Fcγ receptors RI or RIV *in vitro*.

**Discussion:**

The data presented here demonstrate the generation, characterization, and potential of bispecific antibodies targeting two distinct glycoproteins to enhance neutralization of herpes simplex virus compared with monoclonal antibodies. BsAbs offer a potential avenue for herpes therapeutics, but their design and mechanism of action require careful consideration for optimal efficacy. Engineering different formats of BsAbs will not only enable optimal protective and therapeutic outcomes but also aid in the study of the spatial relationships between key glycoproteins involved in HSV infection. We propose that targeting distinct steps of the fusion cascade will yield a BsAb that is highly effective.

## Introduction

Approximately 67% of the global population under the age of 50 is infected with herpes simplex virus type 1 (HSV-1), and 13% of individuals aged 15–49 have herpes simplex virus type 2 (HSV-2) (1). After the initial primary infection of the skin or mucosa, HSV infects sensory nerve cells and is transported to the trigeminal ganglia (HSV-1) or dorsal root ganglia (HSV-2), where it establishes latency (2). Specific triggers [such as fever, stress, and viral infection) can lead to viral reactivation. The infectious virus is transported back to the original site of infection to produce a lytic infection and facilitate transmission (reviewed in (3)]. HSV-1 and HSV-2 are transmitted through contact with an infected person’s lesions, mucosal surfaces, or oral and genital fluids, leading to either asymptomatic infection (4, 5), or less often, to painful blisters (6). Infection with HSV-2 increases the risk of acquiring and transmitting HIV (7). For pregnant women, HSV infection poses a serious risk to the newborn, often resulting in significant illness, death, or long-term complications for infants that survive (8, 9). HSV recurrences in immunocompromised patients are particularly problematic, due to their increased frequency and severity, which may require longer and higher-dose treatment (10, 11). Despite numerous efforts to develop an HSV vaccine to prevent these serious illnesses, none have succeeded. Acyclovir is currently the primary treatment, but morbidity remains high, particularly associated with disseminated disease (12). Since HSV infections are lifelong, optimizing treatment and prevention strategies is crucial.

Passive transfer of HSV-neutralizing antibodies in animal models (13, 14) supports the ability of Mabs to offer both protective and therapeutic benefit, while placental transfer of HSV antibodies protects neonatal mice and humans (15–19). Current therapeutic strategies have focused on anti-HSV monoclonal antibodies (Mabs), with ongoing clinical trials evaluating the therapeutic efficacy of anti-gD Mabs HSV8 (20) and UB-621 (clinical trials NCT02346760, NCT03595995, NCT04714060, NCT04979975) and anti-gB HDIT101 (NCT04539483, NCT04165122). Here, we explore additional antibody-based therapies for HSV that leverage bispecific antibodies (BsAbs) engineered via recombinant DNA technology. Unlike Mabs that target a single antigenic epitope, BsAbs integrate antigen-binding domains from two distinct Mabs into one structure. This design allows BsAbs to simultaneously target two epitopes of the same antigen or even two separate antigens. This capability addresses the issue of antibody resistance often encountered with Mabs and simplifies production by enabling the creation of a single molecule rather than combining two separate Mabs for cocktail therapy, potentially providing a more effective, streamlined treatment approach.

Several BsAbs have been approved for cancer-related treatments (21), and BsAbs targeting viruses, such as those neutralizing SARS-CoV-2 variants (22, 23) and mpox (24), have shown encouraging results. Recombinant DNA technology bypasses the limitations of hybridoma technology, such as low fusion efficiency between B lymphocytes and myeloma cells and reliance on experimental animals. It facilitates the development of various antibody formats, including single-chain variable fragments and nanobodies.

HSV entry into cells relies on four glycoproteins: gD (the receptor-binding protein), gHgL (fusion modulator heterodimer), and gB (the fusion protein). Disabling one or more of these glycoproteins with antibodies can prevent infection (25–30). We previously demonstrated that combinations of two Mabs, targeting gD and gHgL, offer additive or synergistic advantages (31). Furthermore, we designed a highly effective BsAb targeting two non-competing epitopes on gD (32). We used a similar strategy here to design an antibody targeting epitopes on two glycoproteins, gB and gD, rather than two epitopes within the same glycoprotein (gD). Although mechanistically the gB and gD glycoproteins are located at either end of the fusion cascade, they are likely positioned close to each other on the viral envelope (33–36).

To design a BsAb that exploited the non-overlapping roles of gD and gB in the HSV entry and fusion cascade, we engineered two bispecific constructs: MC2/C226sc (IgG and scFv-Fc format) and BD Tetra (a single-chain fusion linked to the heavy chain of MC2). Both formats demonstrated enhanced activity in fusion inhibition and viral neutralization assays, as well as the ability to bind target epitopes in gB and gD simultaneously. In addition, both formats bound mouse Fcγ receptors RI and RIV, supporting the possibility of their Fcs to contribute to protection through antibody-dependent cellular cytotoxicity and phagocytosis. To further understand their mechanism of action, we generated other recombinant forms of the MC2/C226 BsAb in which the size of the paratopes was either increased (both MC2 and C226 as full Fabs) or decreased (both paratopes as single chains). We found that the efficacy of a BsAb depends on the individual properties of the Mabs and the size of the BsAb as a whole. The panel of BsAbs we generated will enable us not only to develop new therapeutic reagents against herpes simplex virus diseases but also to study the spatial relationships and distance between key glycoproteins involved in HSV infection, potentially contributing to future therapeutic strategies.

## Results

### Screening mouse neutralizing Mabs for blocking cell-cell fusion

**Figure 1:**
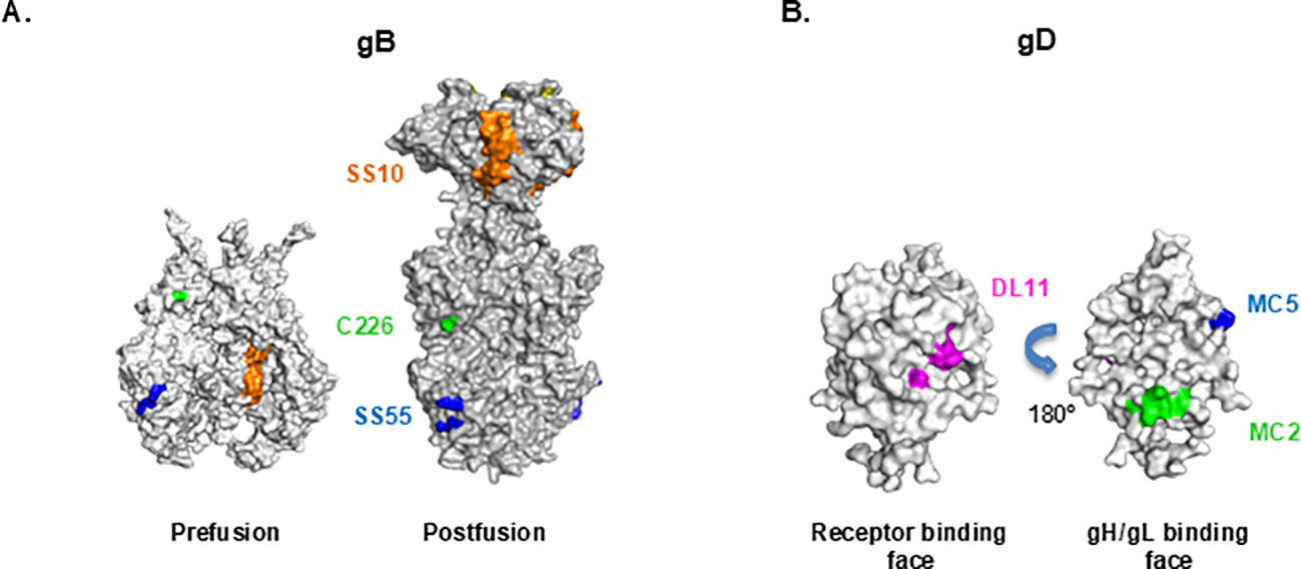
Crystal structures of gB and gD and epitope location for neutralizing monoclonal antibodies. The epitope location of sentinel monoclonal antibodies (colored) used in this study is indicated on the crystal structure of pre-fusion cryo-EM model (PDB: 6Z9M) and post-fusion crystal structure (PDB: 3NWF) of gB_1_(730t) **(A, B)** gD_1_ (285t) (PDB: 2C36).

**Figure 2:**
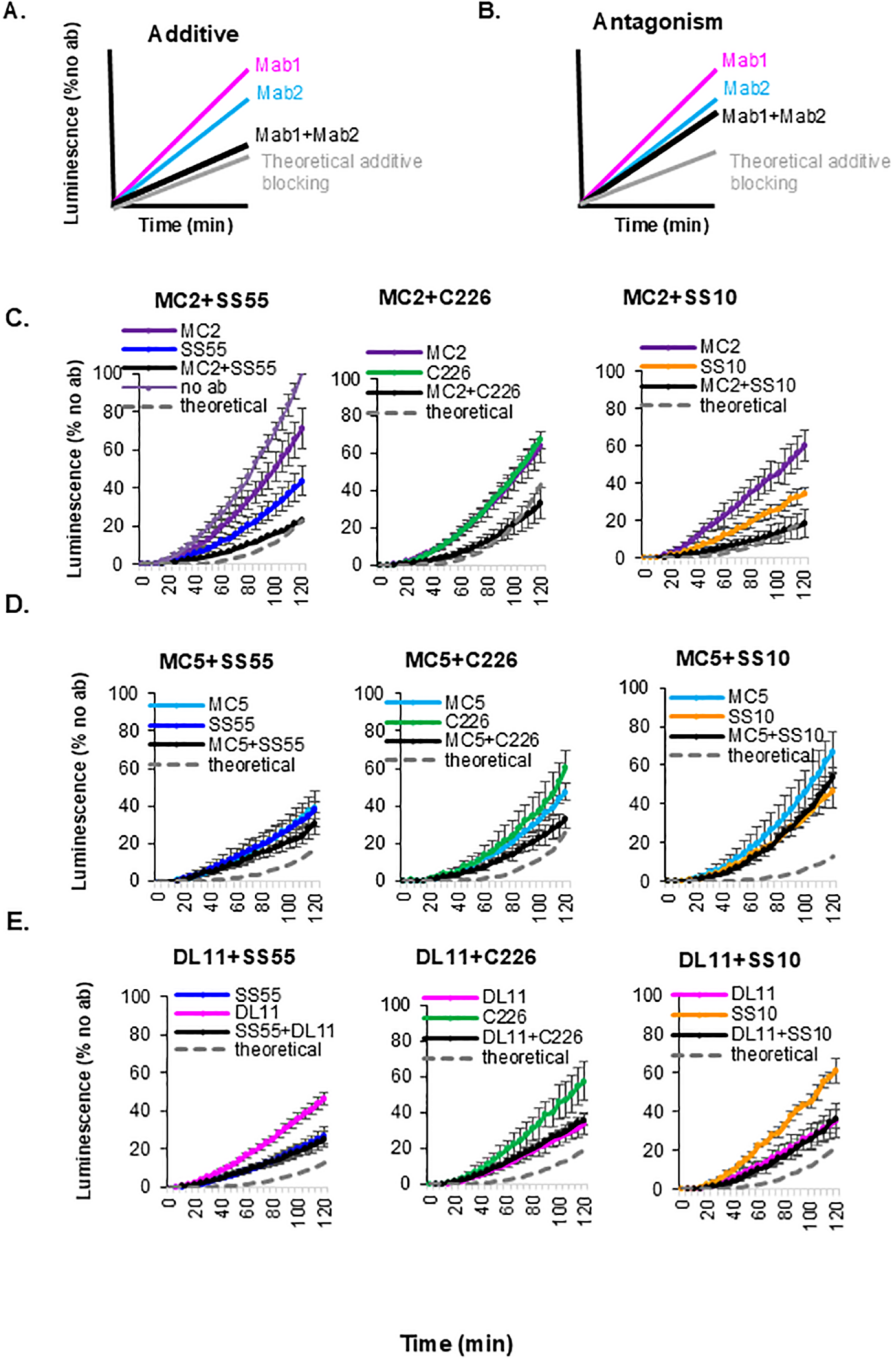
Effect of combinations of gD and gB murine Mabs on cell-cell fusion. Two theoretical outcomes are expected when combinations of non-competing antibodies (colored curves) are used to block cell-cell fusion: additive effect **(A)** when the combination of Abs (black) is as good as a theoretical, additive curve (gray), or antagonistic **(B)**, when the combination is only as good as the more active Ab. Pairs of gD and gB Mabs antibodies (5ug/ml each) were evaluated over 2h. **(C)** MC2+gB Mabs. **(D)** MC5+gB Mabs. **(E)** DL11+gB Mabs. The Bliss independence model was used to evaluate the effects of combinations of Mabs. A theoretical additive curve (gray dashed lines in all graphs) was calculated based on the ability of each Mab to inhibit fusion. Graphs derived from four experiments with standard deviations.

We set out to develop a BsAb targeting epitopes on two glycoproteins, gB and gD, consistent with our prior approach to generate a highly effective BsAb targeting two non-competing epitopes in gD (32). To identify IgG candidates for BsAb design, we screened pairs of gD- and gB-neutralizing Mabs for their ability to block cell-cell fusion. For gD, we chose Mabs that bind distinct epitopes on different faces of the protein and block different functions: binding to the nectin-1 receptor (DL11), interaction with gH/gL (MC5), or stabilization of the gD-gH/gL complex (MC2). For gB, we chose SS55 (blocks insertion of fusion loops into membranes), C226 (interferes with gB-gH/gL interaction), and SS10 (blocks binding to a cellular receptor) (**Figure 1**; **Table 1**). The goal was to identify heterologous pairs of Mabs that have an additive or synergistic effect on inhibiting cell-cell fusion. Because the gD and gB Mabs do not compete for binding and block different steps in the fusion pathway, the contribution of the two Mabs to inhibiting fusion was evaluated using the Bliss Independence model (32, 37–41). This model assumes that two compounds (here, antibodies) with independent binding sites and independent mechanisms of action will not interfere with each other, but both contribute to the overall result. Although the model accounts for four possible outcomes (additive, synergistic, indifference and antagonistic), we simplified this classification into two categories: 1) “synergistic” (**Figure 2A**) where each antibody binds to distinct functional sites, acts independently of each other and both contribute to the final effect; the effect of the combination closely resembles or is better than the calculated theoretical curve and 2) “antagonistic” (**Figure 2B**) for combinations that are only as effective as when one of the Mabs is used alone (location and overall mechanisms of action is the same.

**Table 1:** Properties of neutralizing gB and gD antibodies used in this study.

Glycoprotein	Antibody	Specificity	Epitope/residues	Isotype (Hc/Lc)	Function
gB	SS55	common	199,203,335 (25)	G1/kappa	Fusion loop insertion
	C226	common	419 (82)	2b/kappa	gB-gH/gL interaction
	SS10	common	650-660 (82)	G1/kappa	Receptor binding
gD	MC2	HSV-2	64,67,243,245,246,248 (40, 64)	G1/kappa	gD-gH/gL stabilization
	MC5	common	54,75-79 (40)	G1/kappa	gD-gH/gL interaction
	DL11	common	38,132,140,222-224 (70)	2a/kappa	HVEM binding

The inhibitory effect of two Mabs was determined using a modified split luciferase assay (SLA) (42–45). Effector cells (expressing gB, gD, gH/gL, and half of the split renilla luciferase) were pre-incubated with each Mab. Target C10 cells carrying nectin-1 were transfected with the other half of the renilla luciferase. Fusion was triggered by the addition of C10 target cells to the effector cells. The reconstitution and production of luciferase (as a readout for fusion) were monitored in real time. The blocking activity of the different antibodies was normalized using the “no antibody” samples to reflect no blocking.

Because each Mab blocked a distinct step in the fusion cascade, we expected that all combinations would have at least an additive activity. That was indeed the case for gD Mab MC2 combined with the gB Mabs (**Figure 2C**; compare black and colored curves). However, when the same gB Mabs were mixed with either MC5 (**Figure 2D**) or DL11 (**Figure 2E**), all the combinations were only as active as the stronger Mab (except for MC5+C226 which showed a modest additive effect), suggesting that all these combinations were antagonistic. Based on these results, we conclude that combinations MC2+SS55, MC2+C226, and MC2+SS10 are preferred candidates for bispecific antibody design. We chose the MC2+C226 pair for further studies.

### Isotyping, sequencing, and cloning

**Figure 3:**
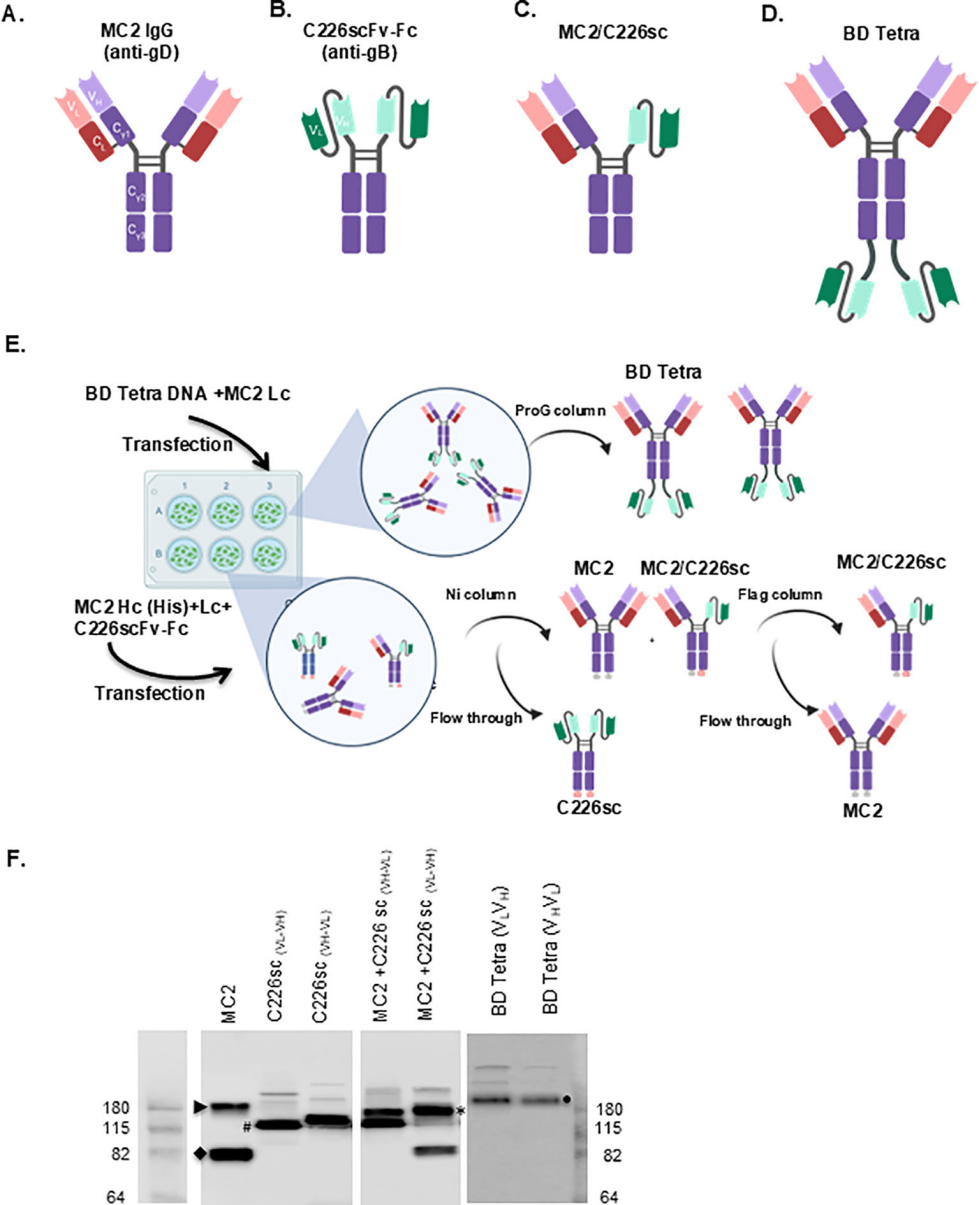
**(A)** schematic representations of the different forms of antibodies. Immunoglobulin G (IgG) is composed of a fragment crystallizable (Fc) region and two fragment antigen binding (Fab) arms. The Fab portion contains the constant domains of the heavy (C_H_) and light (C_L_) chains. The variable domains of the heavy (V_H_) and light (V_L_) chains (Fv) define the antigen-binding site (paratope). **(B)** a single chain antibody (scFv) is composed of the V_H_ and V_L_ domains (Fv region) connected by a short peptide linker to the Fc. **(C)** a bispecific antibody (BsAb) has two distinct paratopes and thus, capable of binding two different epitopes within the same antigen or two different antigens. **(D)** BD tetra has two single chains connected to the heavy chains by a linker. **(E)** schematic representations of expression and purification of BsAbs. 293T cells were transfected with plasmids encoding for MC2 light chain (Lc) and the modified MC2 heavy (Hc). The supernatants were passed through a ProG column to purify BD tetra BsAb. To produce MC2/C226sc, cells were transfected with the Hc and Lc of MC2 as well as the C226scFv-Fc. Supernatants were sequentially passed through nickel and flag columns. **(F)** western blotting. Bands of the different species of recombinant antibodies are readily detected in the supernatant of 293T cells, depending on which plasmids were transfected: MC2 IgG (arrowhead); C226scFv-Fc (hashtag); MC2/C226sc (star); BD tetra (full circle). Cartoons were generated using Biorender (https://app.biorender.com).

Murine hybridoma cells expressing antibodies were isotyped (**Table 1**) and sequenced. The DNA sequences of the heavy (Hc) and light chains (Lc) were cloned into pcDNA3 expression vector. To produce an MC2+C226 bispecific antibody, we left MC2 as an IgG (**Figure 3A**). We designed C226 (**Figure 3B**) as a single-chain antibody (scFv-Fc) to avoid light-chain mispairing during antibody production in tissue culture (46). We generated two versions of C226scFv-Fc: one where the heavy chain variable region (V_H_) was linked via a Gly-Ser linker with the light chain variable region (V_L_) (C226sc(V_H_V_L_)) and the second where the order of the two domains was switched (C226sc(V_L_V_H_)). The co-transfection of MC2 heavy and light chains, as well as the C226scFv-Fc DNAs, leads to the formation of a bispecific antibody, MC2/C226sc (**Figure 3C**), as well as the single IgGs, MC2 and C226sc. For purification, we added a 6xHis tag to the MC2 Fc and a Flag tag to C226scFv-Fc. To reduce the number of DNA constructs that need to be transfected, we designed another recombinant antibody (rAb) in which the C226sc-Fv was cloned at the C-terminus of the MC2 heavy chain. The C226sc portion of this construct was also designed with the C226 V_H_ and V_L_ in different orders. By co-transfecting this modified heavy chain along with the MC2 light chain, another version of the bispecific could be generated, which we named BD Tetra, with the gD antibody shown on top, and gB on the bottom (**Figure 3D**). All antibodies were designed using mouse IgG2a Fc kappa light chain.

### Generation of recombinant antibodies

293T cells were transfected with the heavy and light chains of MC2 and/or C226sc to generate the MC2 IgG, C226scFv-Fc, and MC2/C226sc, or the modified MC2 Hc linked to the C226sc-Fv and MC2 Lc to produce BD Tetra (**Figure 3E**). Supernatants were collected and tested for antibody production by western blotting using an anti-mouse secondary Ab. The single rAbs were detected at the expected molecular weight of ~150 kDa for MC2 IgG (arrowhead in **Figure 3F**) and ~110 kDa for C226scFv-Fc (hashtag). An ~80kDa band, which we believed to be denatured MC2 Hc, was observed in some supernatants. This antibody species was removed during the purification process of both MC2 and MC2/C226sv (**Supplementary S1**). The supernatants from cells co-transfected with MC2 and C226sc plasmids (either C226sc(V_H_V_L_) or C226sc(V_L_V_H_)) contained two major products: one running at ~130kDa (asterisk), the expected size of a MC2/C226sc BsAb, and the other running at ~110 kDa corresponding to either C226sc(V_H_V_L_) when cells were co-transfected with MC2+C226sc (V_H_V_L_) or free MC2 Hc (arrow head) in the samples from the MC2+C226sc (V_L_V_H_) transfection. Supernatants from cells co-transfected with the modified MC2 Hc and Lc contained only one major band of ~200 kDa corresponding to the expected size of BD Tetra Abs (circle). For simplicity, we chose to further characterize the BsAbs containing C226sc (V_H_V_L_). The MC2/C226sc BsAb was purified through Nickel and Flag columns. For BD Tetra Abs, we used a ProG column. All rAbs were pure, ran at the expected molecular weight, recognized soluble gB and gD by western blotting, and could simultaneously bind both target proteins by biosensor (**Supplementary S1**).

### Effect of recombinant antibodies on cell-cell fusion

**Figure 4:**
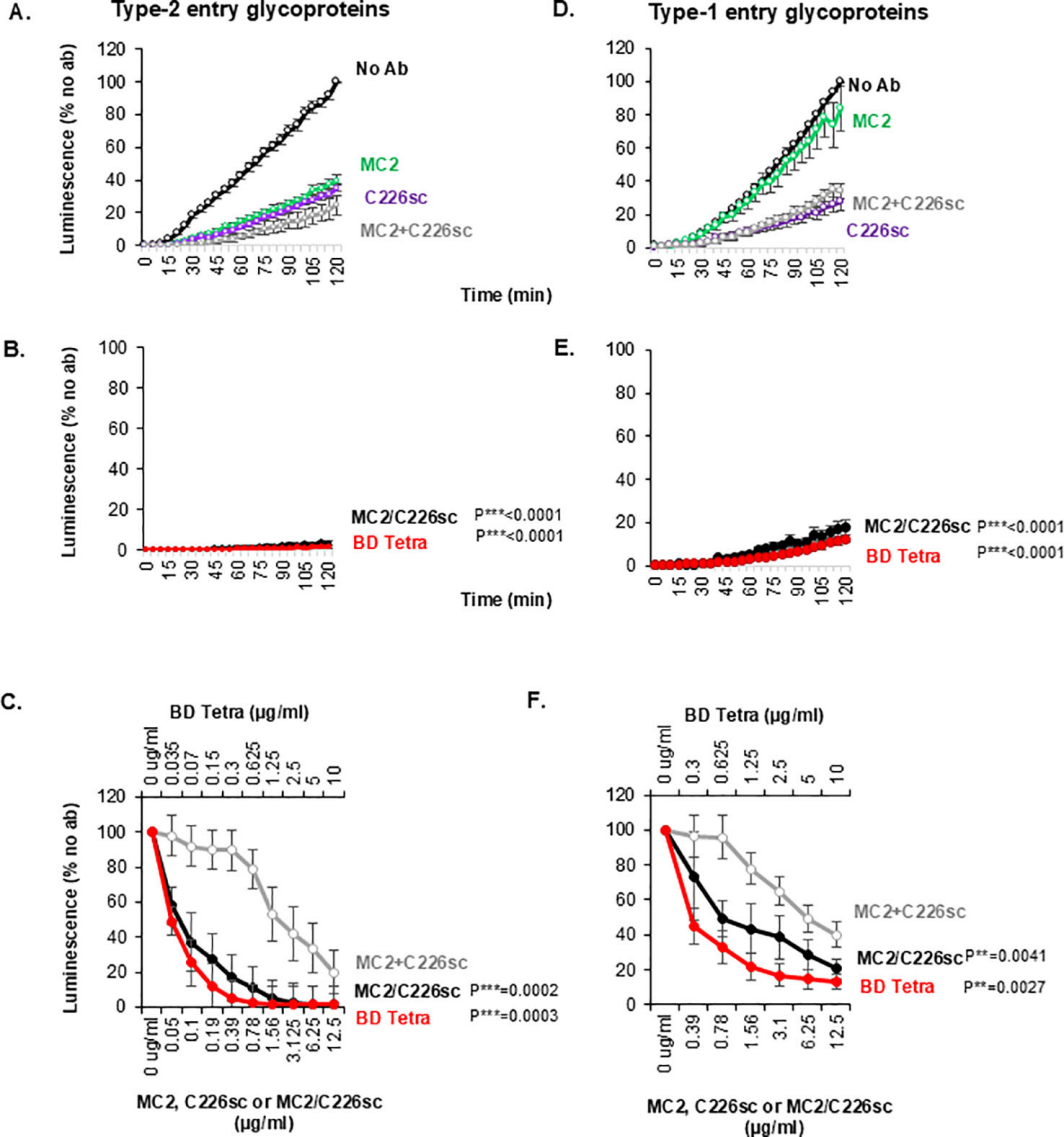
Blocking of cell-cell fusion by rAbs when fusion is mediated by type 2 **(A–C)** or type 1 **(D–F)** glycoproteins. Effect of monospecific antibodies MC2 (green), C226sc (purple), their combination (gray), and the BsAbs MC2/C226sc (black circles) and BD tetra (red). (**C, F**) Titration curves of MC/C226sc and BD tetra compared to the combination of MC2+C226sc. Curves were derived from fusion levels measured at the 2h timepoint in the presence of antibodies at the indicated concentrations. To account for the different molecular weights and ensure similar active units, the concentrations are shown on two x-axes: MC2/C226sc and the combination are shown on the lower x-axis. The BD Tetra concentrations used are shown on the upper x-axis. Averaged data from three independent experiments. Standard deviations are shown. The activity of BsAb at each data point was compared against the combination of single IgGs at the equivalent point. Statistical significance (*) was determined using a paired two-tailed t-test.

Using the cell-cell fusion assay, we tested the biological activity of the BsAbs on cells expressing HSV-2 glycoproteins. Due to their different MW and to ensure equivalent paratope content, we used 6.25 µg/ml for the single antibodies, 12.5 µg/ml for MC2/C226sc, and 10 µg/ml for BD Tetra. The combination of MC2 and C226sc had at least an additive effect on cell fusion (compare the colored and gray lines in **Figure 4A**), as we observed when combining the individual murine Abs (**Figure 2C**). We expected that both BsAbs formats would have activity at least as high as the combinations. Remarkably, we found that both MC2/C226sc (**Figure 4B**, black curve) and BD Tetra (red curve) completely blocked fusion of HSV-2 entry glycoproteins. We also performed a titration experiment in which cells were incubated with MC2+C226sc, MC2/C226sc, or BD Tetra BsAsb and fusion was monitored for 2h. After normalization to the “no antibody” control sample, fusion levels measured at the 2h timepoint were plotted. Titration of both the BsAbs and MC2+C226sc combination revealed that the BsAbs had a synergistic blocking effect compared to the combination (**Figure 4C**).

As part of our characterization of the recombinant antibodies, we repeated the fusion assay using HSV-1 entry glycoproteins. We hypothesized that: 1) because C226sc is a type common Ab, it would inhibit fusion; 2) because MC2 is a gD_2_-specific Mab, it would not inhibit fusion; 3) the MC2+C226sc combination would only be as good as C226sc alone; and 4) both BsAbs would have the same activity as the combinations and reflect the effect of the gB Ab only. As expected, C226sc blocked fusion by HSV-1 entry glycoproteins (**Figure 4D**, purple curve), MC2 did not (green curve), and as a result, the combination was only as good as C226sc (gray curve). Most surprisingly, BsAbs were more active (synergistic) than the combination (**Figures 4E, F**). This suggested that although MC2 alone or in combination was not active in an HSV-1 background, linking the MC2 and C226 paratopes to the same Fc either increased the activity of the gB Mab in an HSV-1 background or that binding of C226 initiated a series of conformational changes that exposed an MC2-like epitope in gD_1_ that the BsAbs could exploit. Based on these results, we conclude that in a cell-cell fusion assay using glycoproteins from HSV-2, MC2/C226sc and BD Tetra, BsAbs are more effective (synergistic) than the single antibodies or their combination. Furthermore, the synergy is preserved in HSV-1, despite the gD_2_ specificity of MC2.

### Effect of single antibodies, combination of Abs and BsAbs on virus activity

**Figure 5:**
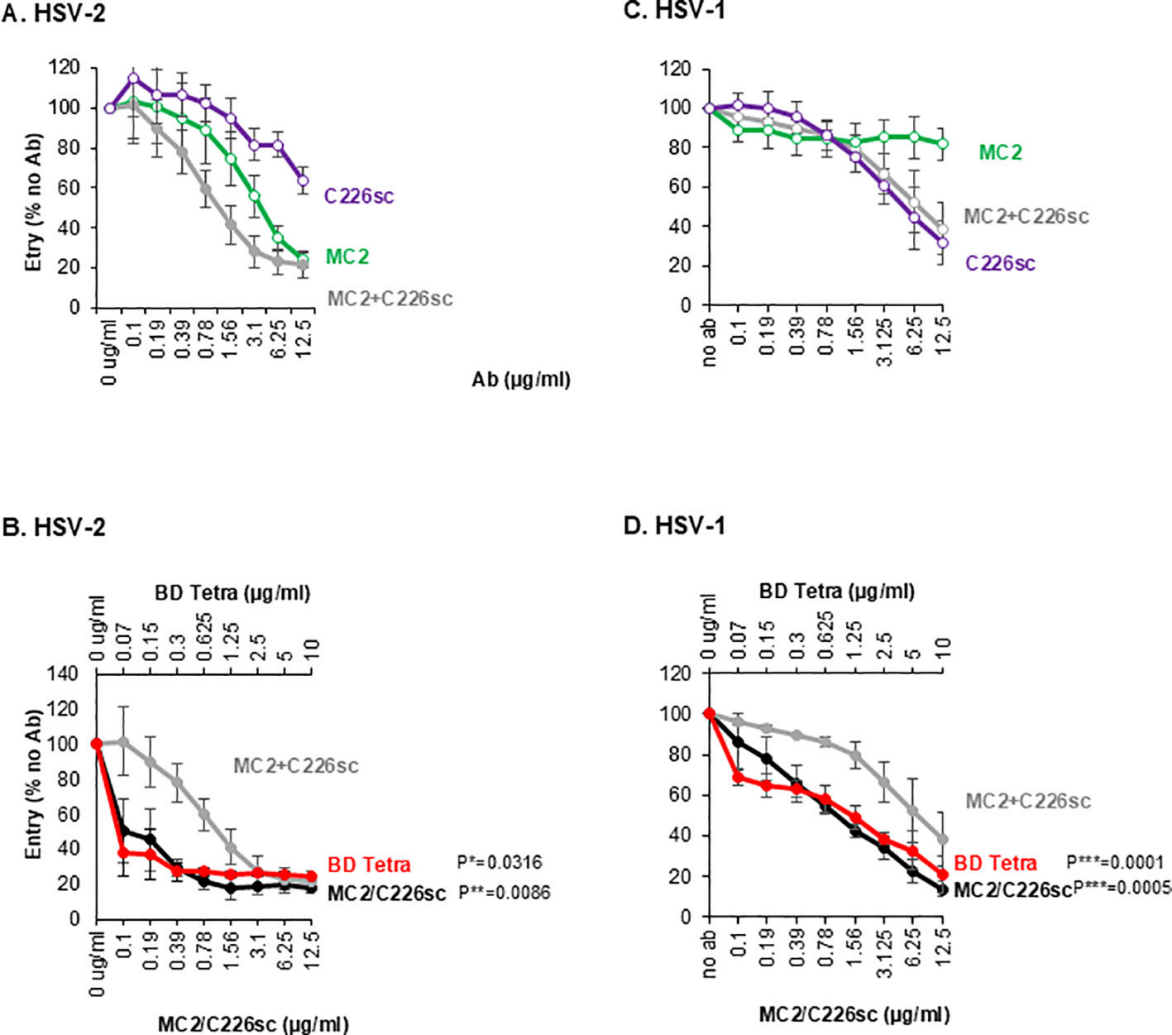
Blocking of virus entry by rAbs. Effect of monospecific antibodies MC2 (green), C226sc (purple), and their combination (gray) against HSV-2 **(A)** and HSV2-1 **(C)**. (**B, D**) titration curves of MC/C226sc and BD tetra compared to the combination of MC2+C226sc. To ensure similar active units, the BsAbs were used at different concentrations: lower x-axis for MC2/C226s and upper x-axis for BD tetra. Averaged data from three independent experiments. Standard deviations are shown. The activity of BsAb at each data point was compared against the combination of single IgGs at the equivalent point. Statistical significance (*) was determined using a paired two-tailed t-test.

Next, we tested the activity of the BsAbs against the virus using a penetration assay. For this, HSV-1 or HSV-2 β-gal reporter viruses were incubated with 2-fold serial dilutions of antibodies. The virus-antibody mix was then added to Vero cells. 6h post-infection, cells were lysed, substrate was added, and β-galactosidase production at 490nm was measured. The activity of the BsAbs (beginning at 12.5 µg/ml for MC2/C226sc and 10µg/ml for BD Tetra) was compared to the activity of the combination or the single Abs (each at 6.25 µg/ml). Much like in the SLA, the combination of MC2+C226sc (**Figure 5A**, gray curve) was better than the activity of the single antibodies (colored curves). Furthermore, the two BsAbs showed synergistic activity against HSV-2, with activity superior to the combinations (**Figure 5B**, compare black, red, and gray curves). As expected, when the same antibodies were tested against HSV-1, MC2 did not contribute to neutralization (green curve in **Figure 5C**). As seen with the fusion assay, although the MC2+C226sc combination (gray) exhibited the same neutralizing activity as C226sc alone, the MC2/C226sc (black) and the BD Tetra BsAb (red) were more active than either C226sc or the combination (**Figure 5D**; **Table 2**). Similar results were obtained when the same antibodies were used in a plaque assay (**Supplementary  S2**).

**Table 2:** Summary of inhibitory activity (IC50) of recombinant mono- and bispecific antibodies against HSV-1 and HSV-2 in a virus entry assay.

Antibody	HSV-2 (μg/ml)	HSV-1 (μg/ml)
MC2	0.8±0.2	>12.5
MC2sc	0.8±0.2	>12.5
C226	1.5±0.2	1.2±0.5
C226sc	12.5±2	12.5±2
MC2+C226	0.8±0.2	1.5±0.5
MC2/C226	0.2±0.1	1.5±0.5
MC2+C226sc	0.8±0.2	>12.5
MC2/C226sc	0.1±0.05	1.5±0.5
MC2sc+C226sc	6±1.5	>12.5
MC2sc/C226sc	1.5±0.5	>12.5
MC2sc+C226	0.8±0.2	12.5±2
MC2sc/C226	1.5±0.2	10±2.5
BD Tetra	0.2±0.1	1.5±0.2

We conclude that just like in the cell-cell fusion assay, BsAbs containing MC2 and C226sc have a synergistic neutralization effect against the virus. Furthermore, they are unique in their activity against HSV-1, exhibiting more activity than MC2+C226sc or C226sc alone.

### MC2/C226sc and BD tetra BsAbs bind mouse Fc gamma receptors the same

**Figure 6:**
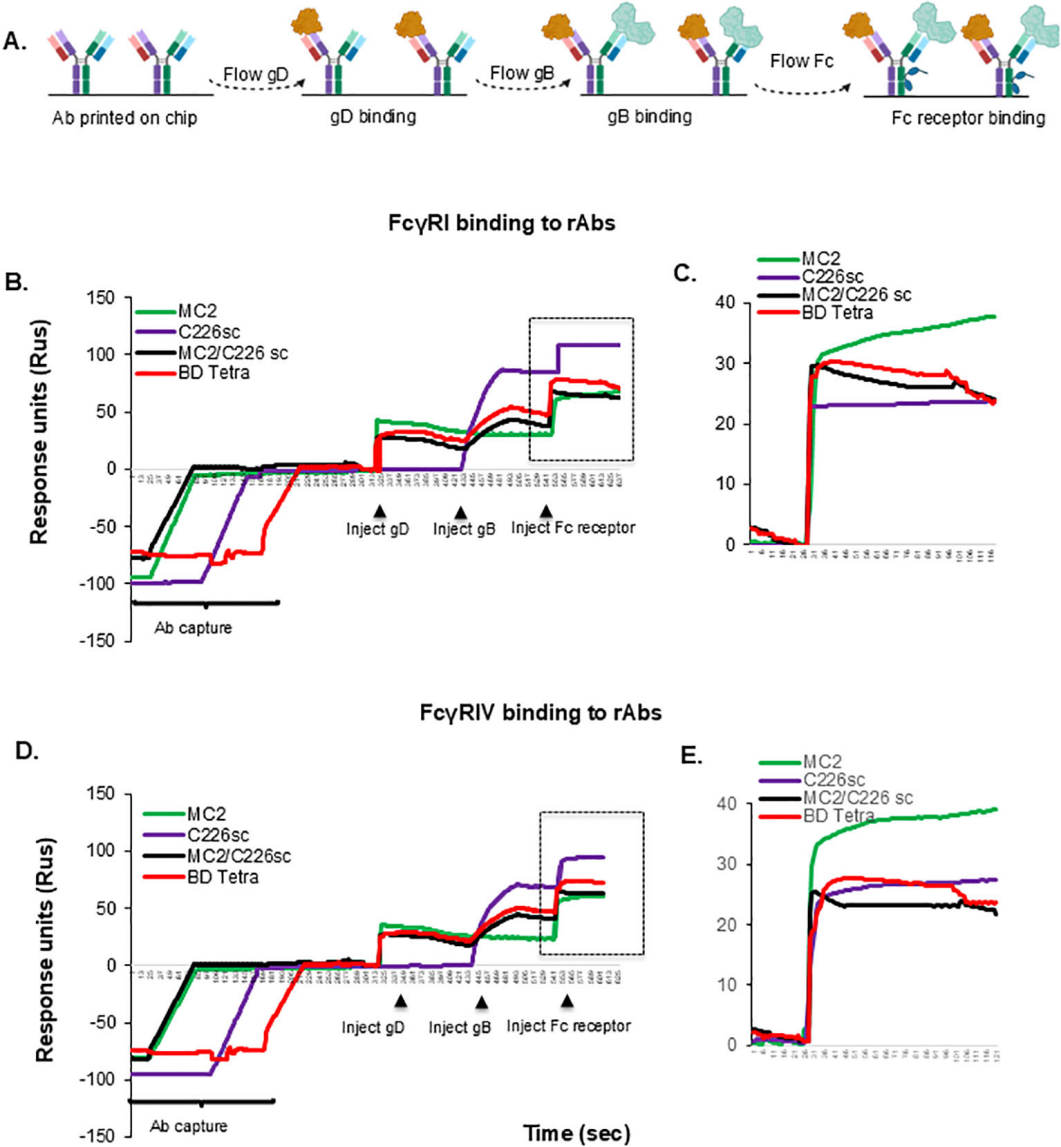
Binding of BsAbs to mouse Fc gamma receptors. **(A)** experiment design. Recombinant antibodies were captured on a ProG chip, followed by the sequential flow of soluble proteins gD and gB. Mouse soluble Fc receptors γI or γIV were flowed over the antibody-antigen complex in separate cycles **(B, D)**. The inset highlights the binding of Fc γRI **(C)** or FcγRIV **(E)** to monospecific or BsAbs. Cartoons were generated using Biorender.

The *in vitro* activity of MC2/C226sc and BD Tetra against the virus suggests that either of these two BsAbs would be excellent candidates for therapy against herpes disease. We tested the ability of the rAbs to bind mouse Fcγ receptors using surface plasmon resonance (SPR). For this, rAbs were immobilized on a ProG chip. We then sequentially flowed purified gD_2_, gB_1,_ and mouse Fc receptors, either FcγRI or FcγRIV (**Figure 6A**). MC2 and C226sc, as well as the BsAbs, bound FcγRI (**Figures 6B, C**) and FcγRIV (**Figures 6D, E**). While there are slight differences in the off rates, the on rates for both receptors are similar. We conclude that the different designs of the BsAbs do not affect the biological and biochemical properties of their paratopes or their Fc domains, and either BsAb would be suitable for immunotherapy.

### The activity of MC2/C226 BsAbs depends on the paratope size

To understand the mechanism of action of the BsAbs, we next addressed whether the observed *in vitro* synergy exhibited by the MC2/C226sc and BD Tetra BsAbs was governed by: 1) an optimal distance between gB and gD on the surface of the virus that would allow for the simultaneous engagement of both paratopes at a crucial time during virus entry; 2) a change in the recognition of gB, gD or both by the BsAbs due to a new presentation of the two paratopes; or 3) increased affinity of the rAbs for target epitopes. To address these questions, we chose the MC2/C226sc BsAb for further manipulations.

**Figure 7:**
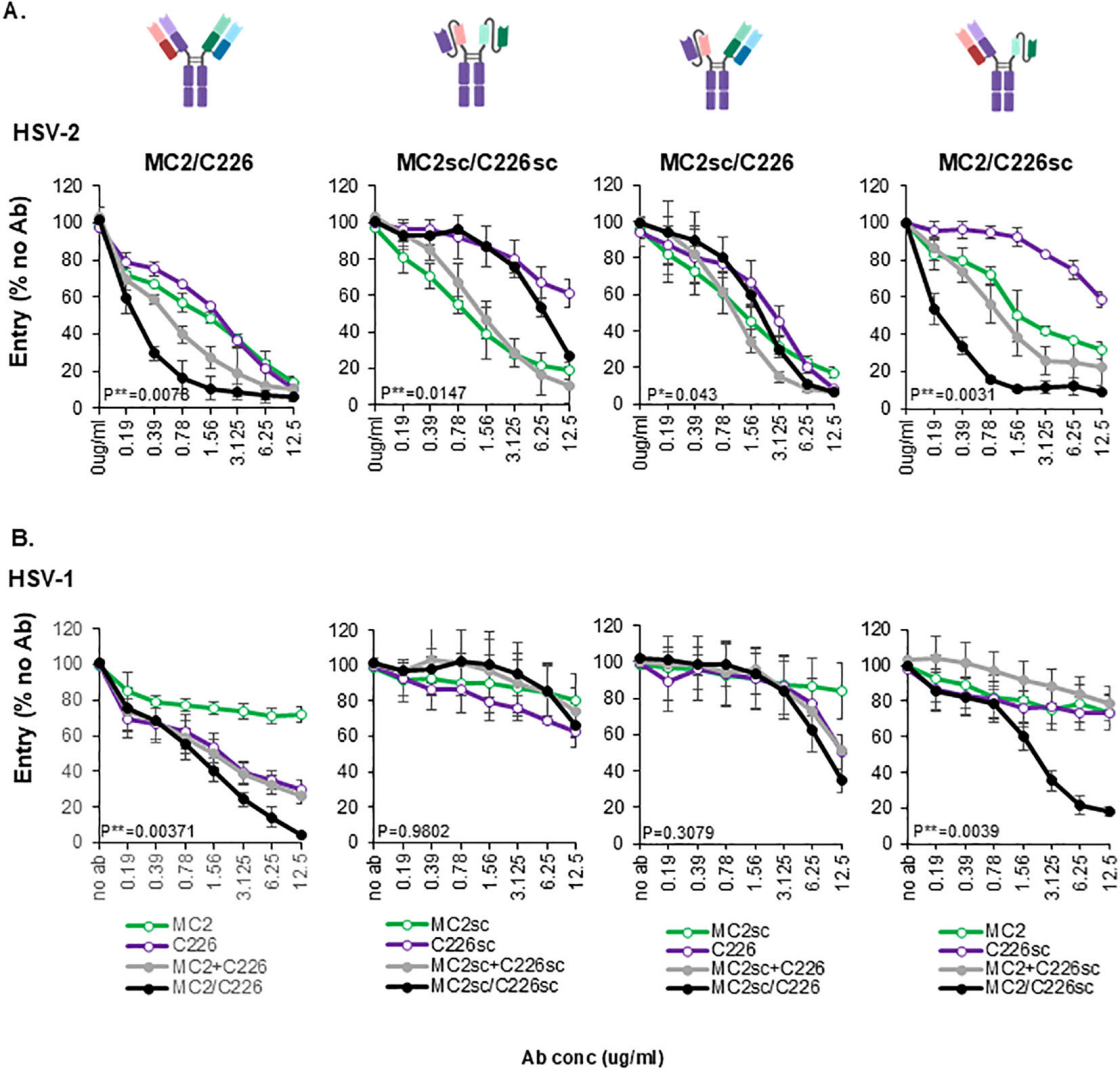
Blocking of virus entry by MC2/C226 BsAbs. Different forms of MC2/C226 BsAbs (black curves)s were tested in an entry assay using HSV-2 **(A)** or HSV-1 **(B)**. The activity was compared to single IgGs (colored curves) and their combinations (gray). Paired two-tailed t-test values of the activity of BsAbs against the combination of single IgGs are shown. Averaged data from three independent experiments. Standard deviations are shown. The activity of BsAb at each data point was compared against the combination of single IgGs at the equivalent point. Statistical significance (*) was determined using a paired two-tailed t-test. Cartoons were generated using Biorender.

To determine if there was an optimal distance between gB and gD that determined synergy, we asked whether the activity of the BsAb would change if the size and reach of the paratopes were increased (by having both arms as full Fabs) or decreased (both arms as single chains). To this end, both MC2 and C226 were designed as either full IgGs or single chains. For MC2sc, the heavy chain variable region was followed by the light chain variable region (V_H_-(G_4_S)_3_-V_L_-Fc). For purification purposes, all MC2 and MC2sc Fcs contained a 6xHis tag at the C-terminus, and C226 and C226sc a Flag tag. By combining the different constructs, we produced MC2/C226, MC2sc/C226sc, MC2sc/C226 and MC2/C226sc BsAbs in 293T cells (**Figure 7A**). The proteins were purified as described in Materials and Methods. In the generation of MC2/C226 BsAb, the light chains can randomly pair with either heavy chain, potentially yielding multiple BsAb variants with different activities and reactivities. However, we have found that >90% of the activity of MC2 and C226 Abs depends on the heavy-chain variable regions. This suggests that light-chain mispairing may not significantly affect the activity of the BsAb population.

To test the activity of these recombinant antibodies against the virus, we used the penetration assay. HSV-2 βGal reporter virus was pre-incubated with 2-fold dilutions of recombinant Mabs. The virus-antibody mix was then added to Vero cell monolayers. After lysis, CPRG substrate was added, and the β-galactosidase activity was measured.

All Abs inhibited virus entry, albeit with different potencies: C226sc showed a marked decrease in activity compared to C226 full-length (compare purple curves in **Figure 7A**), whereas MC2 and MC2sc had similar effects (green curves). The activity of combinations of the different forms of MC2 and C226 was better than that of single forms (gray curves), except for MC2sc+C226sc which was only as active as MC2sc alone. The most potent BsAbs were MC2/C226 and MC2/C226sc. Both MC2sc/C226 and MC2sc/C226sc were antagonistic compared to the respective combinations. Similar results were seen against HSV-1. As expected, there was no neutralization in the presence of MC2 alone (**Figure 7B**, green curve), and the combinations were as active as C226 alone (compare purple and gray curves). The BsAbs containing MC2sc had low activity, followed closely by that of C226 or C226sc. The BsAbs containing full-length MC2 (MC2/C226 and MC2/C226sc) showed synergistic activity compared to the single antibodies and the respective combinations. Similar activity was observed when the BsAbs were tested for plaque-forming activity (**Supplementary S3**). A summary of inhibitory activity (IC50) for all these BsAbs is shown in **Table 2**.

We conclude that the size of the MC2 paratope is essential for the activity of the BsAb against the virus: Abs containing full-length MC2 have a synergistic activity, regardless of the size of the C226 paratope (full Fab or single chain). Despite the type-2 specificity of MC2, MC2/C226 BsAb retains the synergistic activity against both HSV-1 and HSV-2 observed with MC2/C226sc.

### Different-sized versions of MC2/C226 BsAb bind soluble gD and gB as well as monospecific antibodies

Our next question was whether the activity of the different BsAbs is due to changes in their affinity for the target proteins, gB and/or gD, when compared to their parent Mabs. We used biosensor technology (Carterra LSA) to assess binding of the different Ab species to gB and gD and then determine the affinity of each Ab to gD. Murine and recombinant mono- and bispecific antibodies were amine-coupled on an HC30M sensor chip. gD_2_ or gB_1_ purified proteins were injected across the printed antibodies. As expected, recombinant monospecific MC2 and C226 (full length or single chain) Abs showed the same binding profiles to gD and gB as their respective full-length murine versions (**Supplementary  S4**). All versions of MC2/C226 BsAb bound gB and gD similarly (**Supplementary Figure S4**).

For kinetic binding studies of gD, the rAbs were printed on a CRM chip, and 3-fold dilutions of gD_2_ were flowed over the chip surface. For gB binding, we used Biacore and a CM5-Flag chip and 2-fold dilutions of the protein. Binding curves were fitted to a 1:1 binding model. **Supplementary Figure S5** and **Tables 3**, **4** show that all rAbs bound gD with the same affinities. The KD values for gB binding largely followed those of C226 and C226sc: BsAbs containing C226 Fab bound gB with the same affinity as C226 IgG; BsAbs containing C226 as a single chain showed the same affinity as C22scFv-Fc. These data support our conclusion that the binding of the different forms of MC2/C226 BsAbs to gD or gB is not affected by paratope size and is not dependent on valency.

**Table 3:** Kinetics of binding of recombinant antibodies to gD_2_(306t).

Antibody	ka (10^5^ M-1s-1)	kd (10^-3^s-1)	KD (nM)
MC2	1.75	3.03	18
MC2sc	1.5	3.5	19
MC2/C226	2.36	4.13	18
MC2/C226sc	2.73	4.36	17
MC2sc/C226sc	2.26	3.96	17.6
MC2sc/C226	2.1	3.5	21

**Table 4:** Kinetics of binding of recombinant antibodies to gB_1_(730t).

Antibody	ka (10^5^ M-1s-1)	kd (10^-4^s-1)	KD (nM)
C226	3.28	3.67	1.12
C226sc	4.68	2.61	0.57
MC2/C226	1.67	1.89	1.13
MC2/C226sc	3.02	2.65	0.87
MC2sc/C226sc	3.35	1.16	0.34
MC2sc/C226	2.39	3.58	1.5

### MC2/C226 BsAbs bind soluble gB and gD at the same time

**Figure 8:**
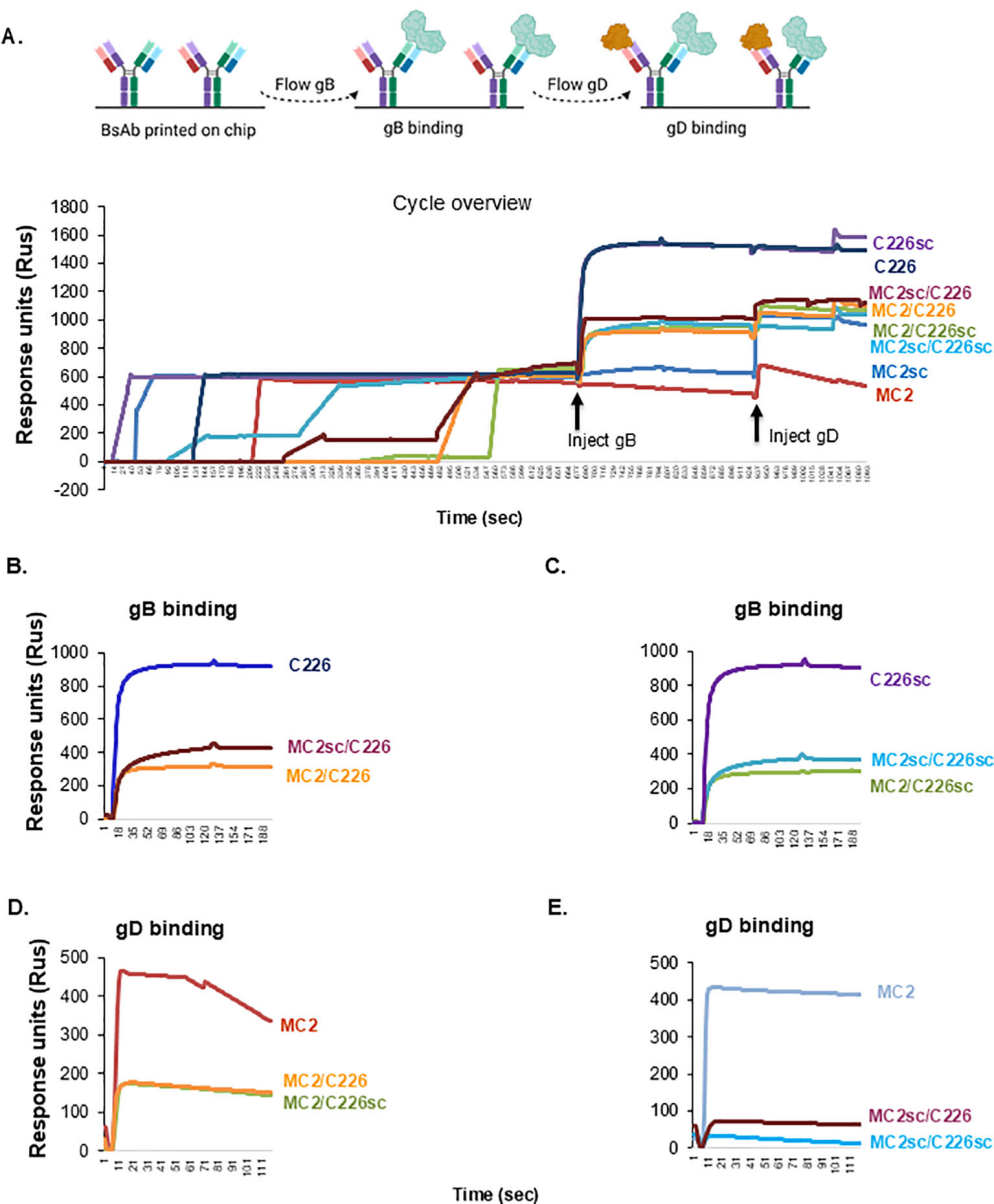
Binding of gB and gD to MC2/C226 BsAbs. **(A)** schematic representation of experimental design and cycle overview. Recombinant antibodies were immobilized on a ProG chip. Purified, soluble gB and gD were flowed sequentially. The increase in Rus after each glycoprotein injection represents an increase in binding. Binding of gB to C226 **(B)** or C226sc **(C)** containing antibodies. Binding of gD to MC2 **(D)** or MC2sc **(E)** containing Abs. Cartoons were generated using Biorender.

The biosensor experiments using a Carterra LSA instrument (**Supplementary S4**, **S5**) showed that the BsAbs bound gD or gB but did not indicate whether the BsAbs could simultaneously accommodate both proteins. To address this, we used a Biacore. After recombinant antibodies were captured, we sequentially flowed soluble gB_1_ and gD_2_. **Figure 8A** shows a diagram of the experiment and an overview of the combined injection cycles with individual gB and gD binding curves shown in **Figures 8B–E**. All BsAbs bound soluble gB, regardless of whether they contained C226 (red and orange curves in **Figure 8B**) or C226sc (cyan and green in **Figure 8C**). As expected, because there is only one active gB-binding paratope, the BsAbs bound less gB than did C226 (dark blue in **Figure 8B**) or C226sc (purple in **Figure 8C**). The biggest difference in protein binding came when we flowed gD over the Ab-gB complexes. gD bound to MC2/C226 (orange in **Figure 8D**) and MC2/C226sc (green). The binding levels of gD to the BsAbs were half of that observed for MC2 (red), as expected. In contrast, the MC2sc/C226 and MC2sc/C226sc showed negligible gD binding compared to MC2/C226 (**Figure 8D**) and MC2/C226sc. These data suggested that, unlike MC2/C226 or MC2/C226sc, BsAbs containing MC2sc were not flexible enough to allow for the binding of both gB and gD, despite their ability to recognize both proteins (**Supplementary  S5**). As the binding capacity of the BsAbs seems to correlate with their biological inhibitory properties, we conclude that BsAbs capable of simultaneous binding of gB and gD (MC2/C226 and MC2/C226sc) will have synergistic activity. At the same time, those that can only bind one target protein at a time (MC2sc/C226 and MC2sc/C226sc) will be antagonistic against virus (**Figure 7**).

#### The distance between gB and gD epitopes is critical for BsAb activity

**Figure 9:**
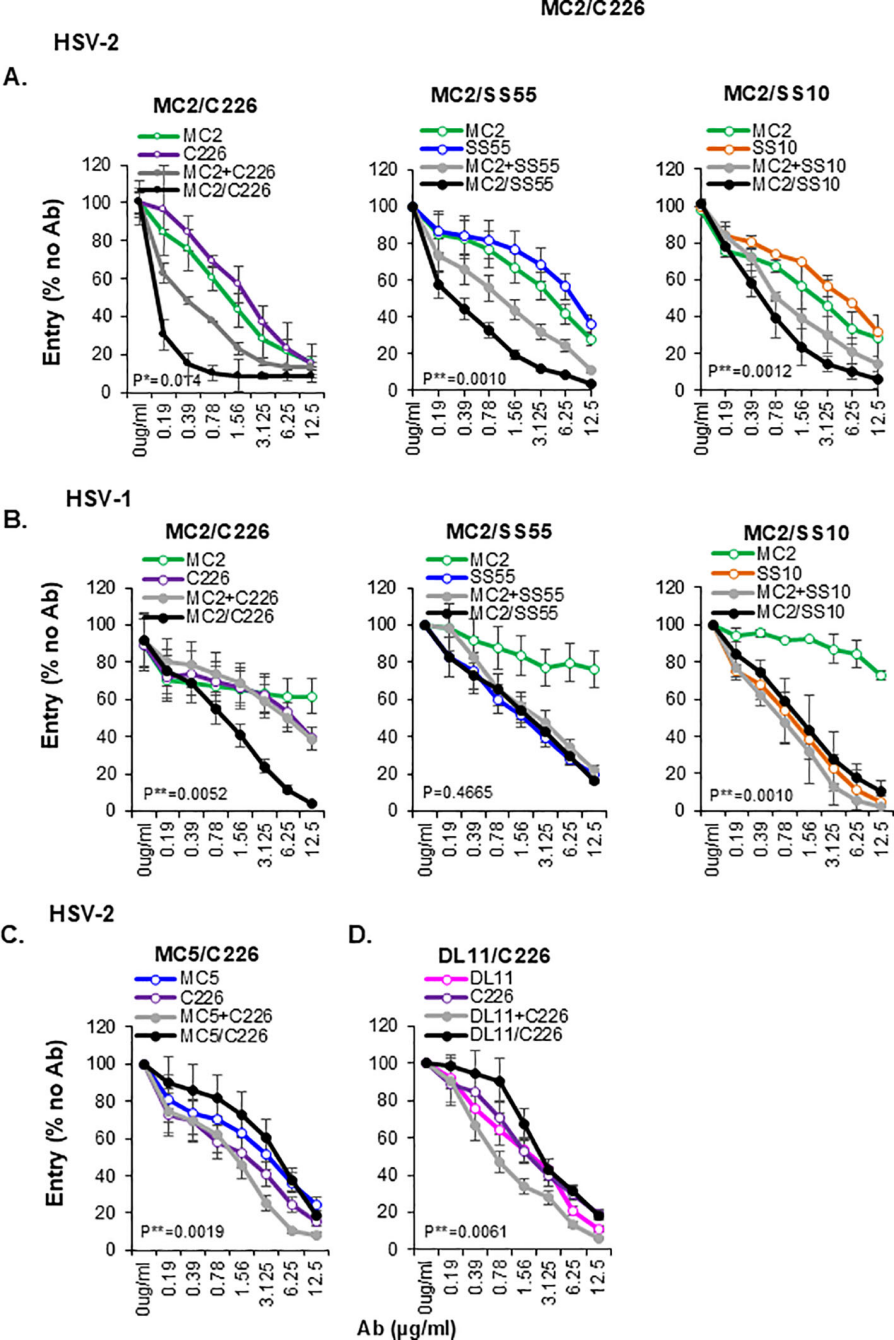
Blocking of virus entry by other gB-gD BsAbs. Effect of MC2 (green), C226 (purple), SS55 (blue), SS10 (orange), combinations (gray), and BsAbs (black) on entry of HSV-2 **(A)** and HSV-1 **(B)** viruses. Effect of MC5/C226 **(C)** and DL11/C226 **(D)** on HSV-2 virus entry. Averaged data from three independent experiments. Standard deviations are shown. The activity of BsAb at each data point was compared against the combination of single IgGs at the equivalent point. Statistical significance (*) was determined using a paired two-tailed t-test.

The remarkable activity of BsAbs containing MC2 and C226 Abs raised the question of whether all BsAbs containing a combination of gD and gB Mabs would be synergistic. The initial screening of pairs of Mabs showed that MC2 had an additive/synergistic inhibitory effect when combined with other gB Mabs (**Figure 2**) and could potentially generate other synergistic BsAb. To produce these BsAb, we co-transfected MC2 Hc (6x His-tagged) and Lc, along with SS55 Hc and Lc (to generate MC2/SS55) or SS10 Hc and Lc (for MC2/SS10). For these preliminary screenings, the BsAbs were purified only on a Nickel column, and the final product was a mixture of BsAb and free MC2. We tested the activity of the new BsAbs against the virus in the penetration assay and used MC2/C226 for comparison. The activity of MC2/SS55 and MC2/SS10 against HSV-2 (black curves in **Figure 9A**) was better than the respective combinations (gray) and comparable to MC2/C226, which was synergistic. However, neither MC2/SS55 nor MC2/SS10 showed synergy against HSV-1, and their activity was no better than that of the strongest Ab (**Figure 9B**).

To determine whether any gD Ab would have a similar contribution to a BsAb as MC2 does, we combined C226 with either MC5 or DL11. Both MC5/C226 and DL11/C226 BsAbs were antagonistic against HSV-2 (black curves in **Figure 9C**). We conclude that although gB and gD are close enough for a BsAb to reach both targets, this requires a specific orientation of the two proteins. Although both the MC5 and MC2 epitopes are located on the same face of gD (**Figure 1B**), only MC2 is at an optimal distance from gB to generate synergistic BsAb against HSV-2 (47).

## Discussion

Bispecific antibodies have emerged as a transformative platform in therapeutic antibody engineering, enabling simultaneous engagement of two distinct antigens or epitopes. Synergistic BsAbs are designed to trigger a biological effect greater than the sum of the effects of the parental monoclonal antibodies. This synergism is often a result of enhanced receptor clustering (48, 49), co-stimulatory signaling (50, 51), improved immune synapse formation (52–55), or modulation of different steps in the same pathway (32, 56).

Computational modeling and structure-guided engineering are playing increasingly important roles in optimizing antibody design for maximal activity with minimal toxicity (57, 58). Despite this, the development of BsAbs presents unique challenges, including manufacturing complexities, stability concerns, and extensive screening of multiple candidate pairs.

Building on our prior work, we sought to design BsAbs that simultaneously target epitopes on two essential HSV glycoproteins, gB and gD. To guide the design, we selected gB and gD-neutralizing Mabs that interfere with distinct steps critical to fusion. While combinations of gD Mab MC2 with any of the gB Mabs were additive, those involving two other gD Mabs, DL11 or MC5, were antagonistic. This suggests that, despite epitope accessibility (these pairs of Mabs target epitopes on two different proteins), there may be greater overlap in their mechanisms of inhibition than we previously thought (31, 32), thereby negating the expected additive function. Identification of pairs of Mabs with a positive phenotype does not guarantee successful BsAbs, as optimal geometry for target engagement is crucial. This requirement applies to all BsAb, regardless of whether the target epitopes are located on the same protein (32, 55) or different proteins. Of the eight BsAbs generated in this study, only three (BD Tetra, MC2/C226sc and MC2/C226) showed an activity at least equal to that of the combination of single IgGs on both HSV-1 and HSV-2. Their IC50 neutralizing activity was comparable to that of HSV8 gD Mab (20) or HD101 gB Mab (59) currently in clinical trials.

One of the key advantages of BsAbs is their capacity to overcome resistance mechanisms associated with monotherapy. In the context of cancer immunotherapy, BsAbs targeting both a tumor-associated antigen and an immune effector molecule, such as CD3 or CD28, can drive more T cell activation and cytotoxicity (60, 61). For viral infections in general, and herpes in particular, targeting multiple epitopes by using BsAbs (32, 55, 56, 62) or combinations of antibodies (63–66) can help overcome viral escape mechanisms. For HSV, neutralizing Mabs stop the infectious cycle. The addition of a second antibody that blocks a different step in viral entry would help neutralize any virus that might escape the first Ab, enhance neutralization, and prevent viral entry by blocking distinct steps in the viral entry cascades.

Antibodies are highly flexible molecules that exist in solution in a variety of structures (67), a property that allows them to crosslink epitopes within approximately 6–12 nm (47, 68). The antibody-antigen interaction begins with the binding of a single paratope. The second paratope will then explore the area until it binds to another epitope (69). The MC2/C226sc and BD Tetra BsAbs showed clear synergistic activity against both HSV-1 and HSV-2, despite MC2 being gD_2_-specific. This suggested that not only were the two glycoproteins positioned at an optimal distance, but that linking the two paratopes to the same Fc resulted in the novel presentation of an MC2-like epitope in gD_1_ that was not achieved through the delivery of the combination of MC2 and C226sc. The re-arrangement of gD_1_ residues to an MC2-like epitope is supported by the knowledge that out of the six residues in gD_2_ essential for MC2 binding, only one is different from gD_1_ (40). Off-target recognition by the BsAb is also a possibility, albeit less likely. The distance between gB and gD appears to be at the lower end of the 6–12 nm range, as reducing the antibody’s reach by using two single chains had an antagonistic effect on virus entry without altering its affinity for the target epitopes. BsAbs containing a full-length MC2 arm (MC2/C226 or MC2/C226sc) were synergistic. In contrast, those with single-chain MC2 arms lost synergy and could not simultaneously bind gB and gD, suggesting that synergy is due to optimal spatial orientation rather than altered affinity for target proteins.

While antagonistic BsAbs might not be desirable therapeutic reagents, they could be valuable tools that inform the spatial organization and orientation of glycoproteins on the virus surface. By systematically combining different gB and gD Abs to generate and characterize BsAbs targeting distinct epitopes on gB and gD, we provide mechanistic rationale for selecting target epitopes. Although MC2/C226 places gB and gD within reach of a BsAb, the orientation of the two glycoproteins is critical. Additional BsAbs combining C226 with DL11, a type-common gD neutralizing antibody that recognizes an epitope situated on the other side of gD, compared to MC2 (40, 70), had an antagonistic effect against virus entry. A recent publication reports a recombinant, synergistic nanobody targeting a DL11-like epitope on gD and a gB epitope (62). This suggests that gD is oriented with the DL11 side facing gB, and that only a BsAb with shorter and smaller paratopes (like the nanobody) could fit within that space.

The reach of a BsAb can be altered in different ways. A popular choice to decrease the size is by generating nanobodies (62). However, if the mechanisms of action of the two antibodies and the distance between epitopes are not fully known, nanobodies may not be as active as expected, and the generation of bivalent homodimers (two binding sites each for gB and gD) may be necessary to improve potency (62). Our decision to reduce the size was to generate single-chain antibodies, with the variable heavy and variable light regions linked by a 15 aa linker. To increase the reach, we used the full-length Fabs or added the single-chain to the C-terminus of a Fab, as we did for BD Tetra. Another alternative is to increase the linker size (71–73). This may affect the immunogenicity of the antibody due to increased O-glycosylation (74), or change its activity and specificity (73). Both increasing and decreasing the size of the antibodies was crucial in determining the mechanism of action of BsAbs.

Structural design, including paratope size, format, and flexibility, is critical for the development of functional BsAbs. Targeting distinct viral glycoproteins involved in sequential steps of membrane fusion and entry can produce synergistic neutralizing activity with implications for broad-spectrum therapeutic antibody design against herpesviruses.

The presence of multiple antigens on the virus envelope makes the development of vaccines or antibody therapeutics to treat herpes disease difficult. We propose that the MC2/C226 or BD Tetra could be effective immunotherapeutic agents for HSV infections. Antibodies could be deployed in several clinically important settings for the management of herpes simplex virus infections. One key application is empiric therapy in patients with suspected severe HSV disease, such as encephalitis or neonatal sepsis, where rapid progression and diagnostic uncertainty necessitate immediate intervention. In this context, Abs could be administered alongside antivirals while awaiting laboratory confirmation, to limit early viral spread and tissue damage. A second scenario involves their use in individuals with drug-resistant infection. Standard drugs target viral DNA polymerase or thymidine kinase, which are prone to mutation-driven escape. As the mechanism of action is entirely independent of enzyme pathways, BsAbs could remain fully potent against strains resistant to acyclovir or foscarnet.

Because Abs can neutralize virus particles and may engage immune effector functions, they could complement the intracellular antiviral activity of nucleoside analogs, potentially leading to more effective viral control. Finally, Abs may be particularly beneficial in immunocompromised individuals who do not respond adequately to standard antivirals or who develop resistant infections, providing an alternative or adjunctive therapeutic strategy. Given the persistently high morbidity associated with severe HSV infections despite antiviral therapy, it is important to evaluate whether incorporating Abs into existing treatment regimens can improve outcomes. Careful clinical studies are needed to determine their impact on survival, disease progression, and long-term complications, as well as to define the optimal timing and patient populations for their use.

Ongoing animal studies are evaluating the therapeutic potential of bispecific antibodies relative to monospecific antibodies in a HSV-1 encephalitis model. The animal models will be used to separate the contributions of the Fc and paratopes to protection by altering Fc effector function. As our understanding of immune and signaling networks improves, humanization of the Fc region and rational design of BsAbs to exploit synergistic interactions will likely expand their clinical utility across a broad range of diseases.

## Materials and methods

### Cells and soluble proteins

Mouse melanoma (B78) and epithelial African green monkey (Vero) cells were grown in DMEM supplemented with 5% FBS and 100µg/ml penicillin-streptomycin. B78-C10 cells, stably expressing nectin-1 receptor, were grown in the same medium as B78 cells, with the addition of 500 µg/mL geneticin. 293T cells were grown in DMEM supplemented with 10% FBS and 100 µg/ml penicillin/streptomycin. HSV-1 KOS tk12 and HSV-2 (333) gJ beta-gal reporter viruses were generously provided by P.G. Spear (75, 76). Wild-type HSV-1 and HSV-2, as well as reporter viruses, were grown and titered on Vero cells. DNAs encoding for gB_1_(730t), gD_2_(285t) and gD_2_(306t) were cloned into pcDNA3. For purification, a 6xHis tag was added to the C-terminus. Proteins were purified from supernatants of transfected CHO cells and purified using Ni-nitriliacetic acid resin column and eluted with 250mM imidazole (77).

Soluble, purified Fc γ RI and Fc γ RIV mouse receptors were purchased from AcroBiosystems (Newark, DE, USA).

### Plasmids

gB_1_ (PEP98), gD_1_ (PEP99), gH_1_ (PEP100), gL_1_ (PEP101) are gifts from Pat Spear (78) and Rluc8_(1-7)_ and Rluc8_(8-11)_ from Zene Matsuda (44, 45). gB_2_ (pTC580), gD_2_ (pTC578), gH_2_ (pTC510), gL _2_(pTC579) were all described previously (42, 79, 80).

### Antibodies

All gD and gB Mabs were previously published: DL11 (81), MC2, MC5 (64), SS10, SS55 and C226 (82).

### Isotyping

All mouse IgGs were diluted to 100 ng/ml and isotyped using Pierce Rapid Isotyping Kit following the manufacturer’s protocol.

### Hybridoma sequencing and generation of antibody-expressing plasmids

Hybridoma sequencing was performed by Genscript (Piscataway, NJ) as described (32). Total RNA was isolated from hybridoma cells and reverse-transcribed into cDNA using isotype-specific anti-sense primers or universal primers following PrimeScriptTM 1st Strand cDNA Synthesis Kit’s manual. Gene synthesis and direct cloning into pCDNA3.1 were done by Genscript. All heavy chains were synthesized as mouse IgG2a isotype with a 6xHis or a Flag tag at the C-terminus, as indicated; all light chains were mouse kappa.

### Expression and purification of recombinant antibodies

2 x 10^7^ 293T cells were transfected with 18 µg of each of the heavy (H_C_) and light chain (L_C_) plasmids using Opti-MEM medium (Gibco) and 180µl of Lipofectamine2000 (Invitrogen). 48h later, supernatants were collected, and cells were refed with Opti-MEM for another 48h. Supernatants from both collections were pooled, clarified by centrifugation, and filtered before dialysis with PBS overnight at 4° C. The monospecific antibodies were purified on a ProG column (Genscript). For bispecific antibodies, the dialyzed supernatants were incubated with “Ni-NTA superflow” nickel resin (Qiagen), with gentle shaking for 24h at 4 °C. After washing with PBS and wash buffer (10 mM imidazole, 20 mM phosphate (pH 7.5), 500 mM NaCl) the Abs were eluted with elution buffer (20mM, 50mM and 250mM imidazole, 20 mM phosphate (pH 7.5), 500 mM NaCl). The 250 mM eluate was dialyzed against PBS. For a two-step purification of BsAb, 0.5 mls of Flag resin (Genscript, Piscataway, NJ) was equilibrated in 1ml TS buffer (500mM NaCl, 10mM Tris in water). The eluate from the nickel column was loaded onto the Flag column. After washing the column with 10 mls of TS buffer, Abs were eluted with 5 mls of alkaline buffer (0.1M Tris, 0.5M NaCl, pH 10.2). The eluate was dialyzed overnight against 1xPBS and then concentrated.

### Western blotting

For evaluation of antibody production: 100 ng of purified antibody were run on a Novex 10% Tris-Glycine gel under “native” conditions (81). Blots were probed with goat anti-mouse or goat anti-human peroxidase and developed using Pierce substrate. For glycoprotein recognition: 50ng of gD_2_306t or 300 ng gB_1_730t were run on 10% Tris-Glycine gels under native conditions. After transfer, nitrocellulose membranes were probed with either supernatant or 1ug purified antibodies as indicated.

### Carterra LSA data collection

Binding of soluble gD2(306t) and gB1(730t) and kinetics of binding of gD2(306t) to recombinant mono- and bispecific antibodies were performed using a Carterra LSA biosensor (Carterra, Salt Lake City, UT, USA), at room temperature. Running buffer was 1X HBSTE (10 mM HEPES, 150 mM NaCl, 3 mM EDTA, 0.005% polysorbate 20, 0.01% Tween 20).

For simple antigen binding: antibodies (mono- or bispecific) were immobilized onto the chip surface by direct amine coupling using a HC30M chip and a 96-channel print-head. First, the chip surface was activated with 100mM N-hydroxysuccinimide (NHS), 400mM 1-ethyl-3(3-dimethylaminopropyl) carbodiimide hydrochloride (EDC) (Cytiva), and 0.1M MES buffer pH 5.5 mixed at a 1:1:1 ratio. Then, antibodies were printed in triplicate at a concentration of 10 µg/mL. This was followed by an injection of 1 M ethanol-amine-HCl (pH 8.5) to quench the chip surface. The antigen was injected using the single flow cell head (SFC) over the chip surface at a 50nM concentration under the following conditions: 1min baseline, 4 min association, 1 min dissociation. Chip surface was regenerated with two 25 sec pulses of glycine pH 2. The collected data were processed using the Binning software (Carterra).

The kinetics of gD binding to murine and recombinant antibodies were performed as described, with the following modifications: murine and recombinant antibodies were immobilized at 0.1, 1, and 10 µg/ml. A three-fold dilution series of antigen was prepared in 1x HBSTE buffer starting at 55nM (for murine MC2, recombinant MC2, MC2sc) or 67nM, as indicated. The antigen was injected using the SFC over the chip surface from the lowest to the highest concentration, without regeneration, under the following conditions: 1min baseline, 5 min association, and 15 min dissociation. The chip surface was regenerated with 10mM glycine pH 2. The collected data were processed using Kinetics software (Carterra).

### Surface plasmon resonance

Binding experiments were carried out on a Biacore 1K+ (Cytiva) at 25 °C. The running buffer for all experiments was 1x HBS-EP+ (10 mM HEPES, 150 mM NaCl, 3 mM EDTA, 0.005% polysorbate 20). 1 µg/ml of the indicated antibodies was immobilized onto a ProG chip (Cytiva). Proteins (gB1(730t), gD2(285t), mouse receptors Fc γ RI or Fc γ RIV) were injected at 30 µg/ml. Glycine pH 1.5 was used to remove Ab-protein complexes from the chip surface until the response signal returned to baseline.

The affinity of gB to recombinant antibodies was determined using a multi-cycle kinetic experiment. 20 µg/ml of each antibody was immobilized to a CM5 chip via an anti-Flag antibody. Two-fold dilutions of gB_1_(730t) protein beginning at 125nM were flowed over with a 30 µl/min flow rate, 120 sec contact time, and 600 sec dissociation time. The chip surface was regenerated to baseline with glycine at pH 1.5 before a new concentration of gB was flowed over. Data was analyzed using Biacore Insight Evaluation software 5.

### Split luciferase assay

B78 and C10 cells were plated in preparation for transfection based on previously described protocols (42, 43). B78 cells plated on a 96-well plate were transfected with 40 ng each gB, gD, gH, gLand Rluc8_(1-7)_. C10 cells were plated on 6-well plates. Each well was transfected with 1 µg of the Rluc8_(8-11)_ plasmid. 24h post-transfection, B78 cells were pre-incubated with Mabs for 1h: 5 µg/ml of each murine antibody (10 µg/ml total when combining two Mabs); 6.25 µg/ml for each recombinant antibody (12.5 µg/ml for combination); 10 µg/ml for BD Tetra. Fusion was triggered by the addition of C10 cells to the B78 cells. Reconstitution of luciferase was monitored for 2h, with readings taken every 5 minutes, using a BioTek plate reader (Agilent Technologies, Santa Clara, CA). Blocking activity of antibodies was expressed as percentage of cell-cell fusion compared to fusion in the absence of Abs (100%).

### β-galactosidase reporter assay for HSV entry

Confluent Vero cell monolayers were grown in 96-well plates and infected at a multiplicity of infection (m.o.i) of 1 with HSV-1 or HSV-2 β-gal reporter viruses. Viruses were pre-incubated with the Mabs at the indicated concentrations for 1h at 37 °C before addition to the Vero cells. After 6h, cells were lysed with 0.1% Nonidet P-40 (Sigma) and CPRG (chlorophenol red-β-d-galactopyranoside, Roche Diagnostic, Indianapolis, IN) was added. The β-galactosidase activity was measured at 595 nm using a BioTek plate reader. β-galactosidase activity indicated successful entry. Antibody blocking activity was expressed as the percentage of virus entry into cells in the absence of antibodies (100%).

### Plaque assay

100 plaque forming units of wt HSV-1 or HSV-2 were pre-incubated with Mabs at the indicated concentrations for 1h at 37 °C. The mixture was added to Vero cells for an additional hour at 37 °C. Monolayers were then overlaid with 1% methylcellulose and incubated with the Mab-virus mixture for 2–3 days, until plaques were visible. Cells were fixed with 5% formaldehyde solution. Plaques were stained with crystal violet and counted.

### Evaluation of the effect of combination antibodies

The Bliss independence model (37) was applied. The activity of each individual Mab was used to calculate a theoretical additive curve using the formula for probabilistic independence:

E_A_ +E_B_(1-E_A_)=E_A_+E_B_-E_A_E_B_.

### Statistical analysis

Two-tailed Student *t* test (GraphPad Prism 10) was used to determine *p*-values.

## Data Availability

The original contributions presented in the study are included in the article/**Supplementary Material**. Further inquiries can be directed to the corresponding author.
