# Supplementary figures and images for: Bispecific antibodies targeting herpes simplex virus glycoproteins B and D
Source: Front Immunol. 2026 May 11;17:1803389. doi: 10.3389/fimmu.2026.1803389 (PMC13199292; doi:10.3389/fimmu.2026.1803389)

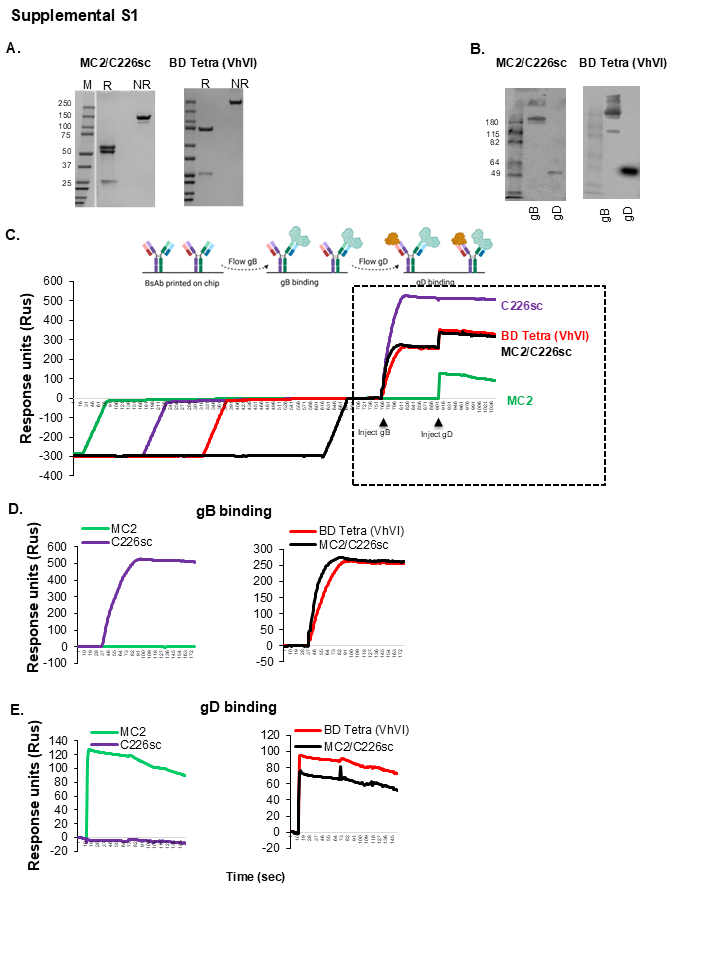

Supplement: Supplementary file 1 [file Image1.tif]

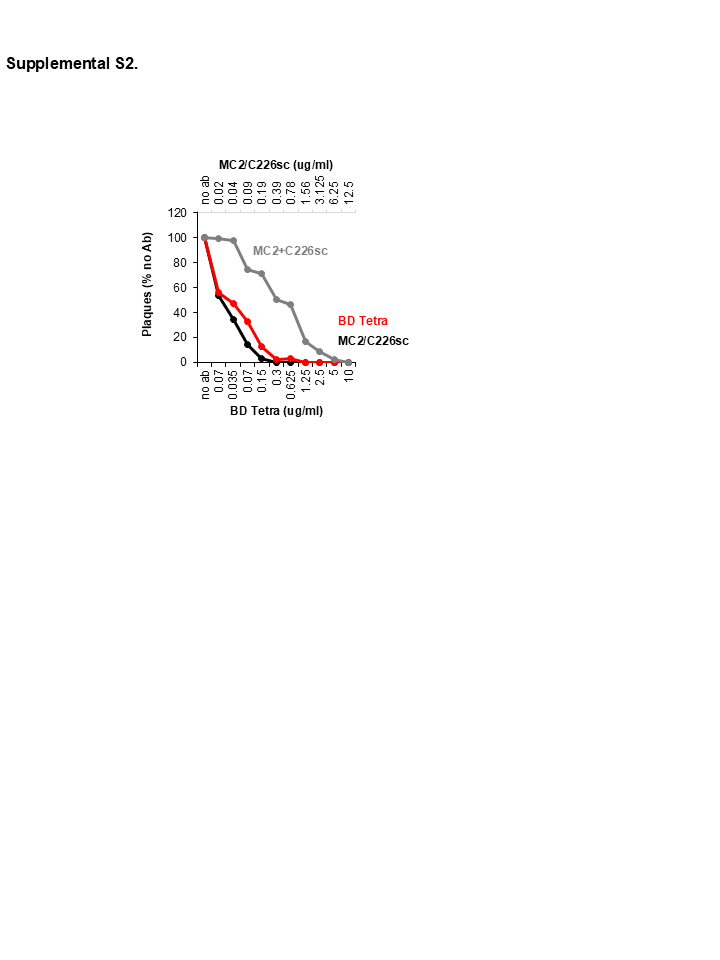

Supplement: Supplementary file 2 [file Image2.tif]

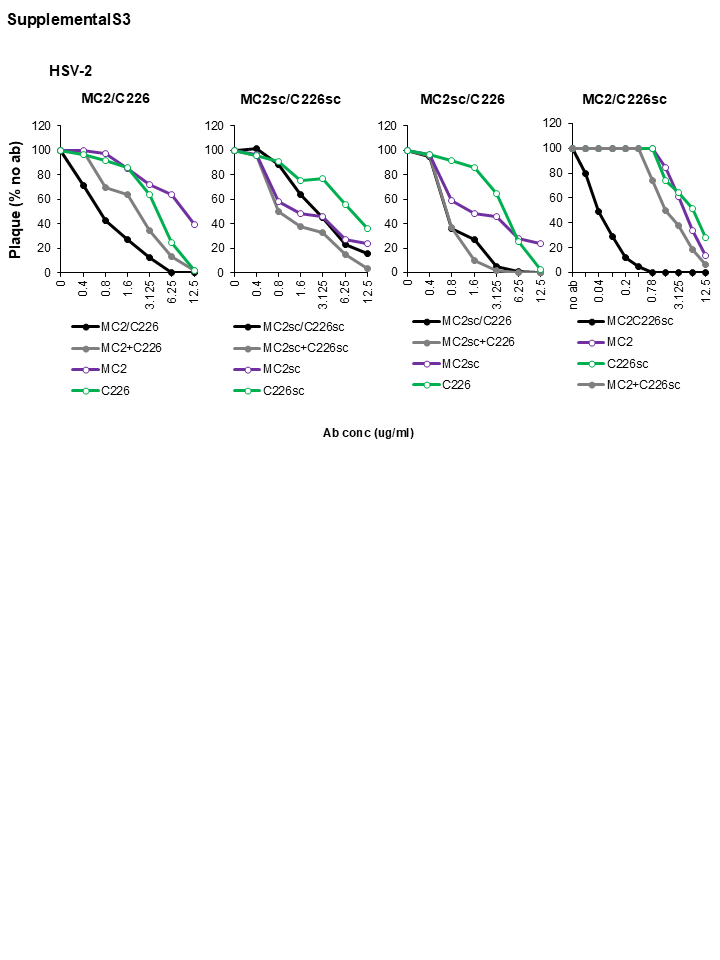

Supplement: Supplementary file 3 [file Image3.tif]

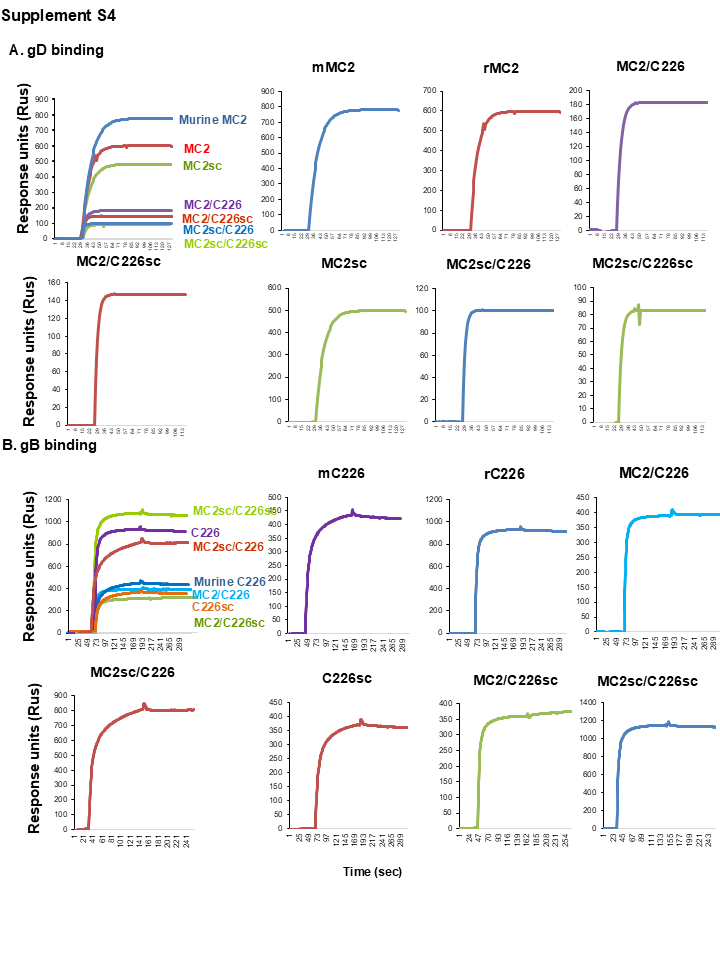

Supplement: Supplementary file 4 [file Image4.tif]

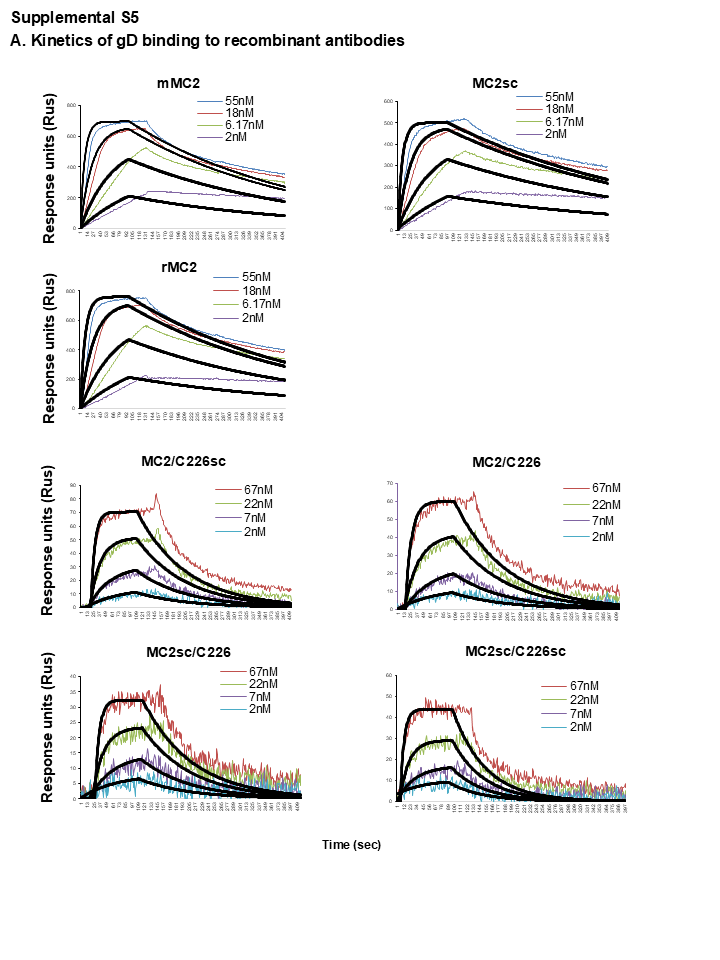

Supplement: Supplementary file 5 [file Image5.tif]
